# Iatrogenic Diversion of Inferior Vena Cava into Left Atrium after Surgery for a Rare Combination of Congenital Heart Diseases

**Published:** 2016-04-13

**Authors:** Feridoun Sabzi

**Affiliations:** Imam Ali Hospital, Kermanshah University of Medical Sciences, Kermanshah, Iran.

**Keywords:** *Heart defects, congenital*, *Congenital abnormalities*, *Vena cava, inferior*, *Heart atria*, *Heart septal defects, atrial*

## Abstract

Atrial septal defect (ASD) is a common congenital anomaly that has low surgical mortality and morbidity. We report a very rare case of a low-lying ASD, combined with the drainage of the inferior vena cava and the left superior vena cava into the left atrium. This combination was associated with an unroofed coronary sinus. We also describe an iatrogenic surgical diversion of the inferior vena cava into the left atrium with its complication. The patient presented with moderate cyanosis and was referred for elective ASD repair. He underwent surgical repair of the ASD after transthoracic echocardiography. Early postoperative right-to-left shunting with cyanosis and hypoxia was associated with abdominal complications. Surgical re-exploration revealed the diversion of the inferior vena cava into the left atrium, which was repaired with a pericardial patch. Peptic ulcer perforation was repaired after abdominal laparotomy. The patient had an uneventful recovery and was discharged home on the 17^th^ postoperative day. One-year follow-up revealed no recurrence of cyanosis or residual ASD on echocardiography.

## Introduction

During fetal life, the Eustachian valve directs the oxygen-rich blood from the inferior vena cava (IVC) toward the foramen ovale and away from the tricuspid valve. The Eustachian valve is an embryonic remnant of the IVC valve. A left superior vena cava (SVC) with an unroofed coronary sinus draining into the right atrium is a common anomaly, but a combination of a prominent Eustachian valve with an atrial septal defect (ASD), anomalous orifice of the IVC in the left atrium, unroofed coronary sinus, and left SVC is extremely rare. In some ASDs, the lower margin of the defect rests at the level of the superior margin of the Eustachian valve of the IVC and, thus, communicates directly into the left atrium. In such cases, there is also an association with a right-to-left inter-atrial shunt.^1^

In the secundum type ASD, the defect is not localized to the area of the foramen ovale but involves the lower part of the septum in the vicinity of the anomalous IVC origin in the left atrium. The inferior margin of the defect is constructed by the superior margin of a prominent Eustachian valve and not by the inter-atrial septum. It is important to recognize this type of defect because if the Eustachian valve is wrongly taken as the lower margin of the defect, the IVC would then be connected completely to the left atrium. The diversion of the blood from the IVC into the left atrium is a serious complication. There have been reports of the intraoperative death of two patients in whom this complication was not corrected.^2^^, ^^3^

## Case Report

An 18-year-old man referred for ASD repair to our hospital. He was moderately cyanotic but reported no chest discomfort, dyspnea, or hemoptysis. He was in New York Heart Association functional class I. On physical examination, the patient had no dysmorphic features but had moderate central and peripheral cyanosis, stage II clubbing with drumstick appearance of the fingers and toes, pulse rate of 90 beats per minute, and blood pressure of 130/70 mmHg. Blood gas analysis demonstrated oxygen saturation of 85% in room air. Cardiac examination showed a normal apical impulse, no right ventricular heaves or thrills, and normal first and second heart sounds. Chest examination was unremarkable apart from lower-limb cyanosis. Laboratory examination revealed serum hemoglobin of 18 g/l, hematocrit of 55%, and normal serum electrolyte levels and renal function. The electrocardiogram and chest X-ray were normal. Transthoracic echocardiography showed normal left and right ventricular functions and dimensions. Also, no tricuspid insufficiency jet was identified from which a systolic pulmonary arterial pressure could be estimated. However, the estimations of the mean pulmonary arterial pressures using acceleration time and pulmonary regurgitation were normal. No persistent left SVC was seen, and nor was there a report of an anomalous drainage of the IVC into the left atrium. Cardiac angiography was not performed to visualize the anatomy of the ASD and IVC. 

The patient underwent ASD repair with bicaval and aortic cannulation. Gross examination revealed that the left SVC drained into the left atrium at the entrance of the left superior pulmonary vein into the left atrium. After an antegrade aortic injection of cardioplegia and the subsequent cardiac arrest and local hypothermia, the right atrium was opened and there was an unroofed coronary sinus to the left atrium, which was treated via extracardiac ligation of the left SVC. Moreover, there was a large low-lying secundum type ASD in the vicinity of an undiagnosed anomalously displaced IVC orifice in the left atrium. The defect was repaired with a pericardial patch, with continuous running suture. The patch was wrongly placed over the upper margin of the Eustachian valve and sutured to the lateral and inferior margin of the defect; this completely diverted the IVC into the left atrium. After repair, the right atrial incision was closed and the aortic cross-clamp was opened. Weaning from cardiac pulmonary bypass was complicated by a low cardiac output and central cyanosis. The volume of ringer lactate and normal saline was increased, but the patient's central venous pressure was not elevated and remained below 5 cm H_2_O.

With a low cardiac output, low arterial saturation, and inotropic drug support, the patient was transferred to the Intensive Care Unit, where he was connected to the ventilator with 100% fraction of inhalation oxygen (FIO_2_). Despite this high fraction of inhalation oxygen, however, blood gas analysis revealed a pressure of arterial oxygenation (PaO_2_) of 56 mmHg and saturation of 70%. Lactic acidosis was treated using a repeated dose of bicarbonate. 

**Figure 1:**
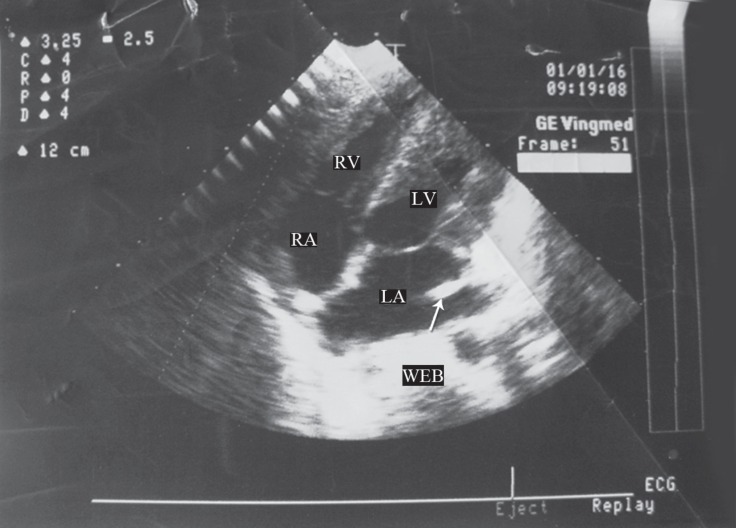
Four-chamber echocardiography view, showing the presence of the Eustachian valve and entrance of the inferior vena cava into the left atrium (arrow)

RV, Right ventricle; LV, Left ventricle; RA, Right atrium; LA, Left atrium

**Figure 2:**
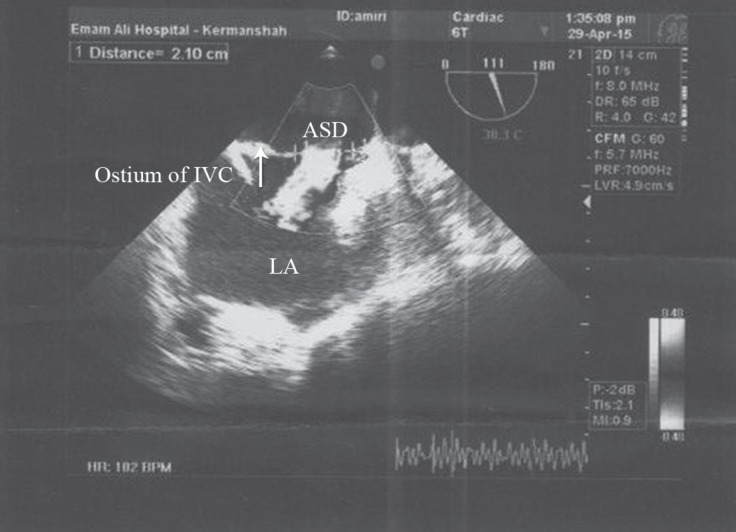
Transthoracic bicaval echocardiography view, showing the web of the Eustachian valve (arrow), the left atrium (LA), and the ostium of the inferior vena cava (IVC)

The following day, when the anesthesiologist tried to wean the patient from the ventilator, the patient's hemodynamic and blood gas analysis was compromised and he complained of severe epigastric pain. He was, therefore, sedated and reconnected to the ventilator. Transthoracic echocardiography illustrated the diversion of the IVC into the left atrium (Figures 1 and 2). The patient underwent reoperation, during which the pericardial patch was removed, the upper margin of the Eustachian valve was excised, and the lower margin of the patch was sutured to the wall of the left atrium below the IVC orifice. After diverting the IVC into the right atrium and opening the aortic cross-clamp, the patient was weaned from cardiopulmonary bypass uneventfully. 

On the following day, the patient was fully conscious and was weaned from the ventilator. Nevertheless, he complained of severe epigastric pain. Physical examination revealed a tense and tender abdomen. In the semi-setting position, abdominal X-ray showed gas-fluid level below the diaphragm. The patient was, consequently, subjected to laparotomy, which revealed a perforated duodenum. The duodenum was repaired and the abdomen was closed. 

The recovery of the patient was uneventful and he was discharged 2 weeks later from the hospital. At one-year follow-up, there was no recurrence of cyanosis or presence of a residual ASD.

ASD, Atrial septal defect

## Discussion

Byor k et al.,^1^ Alabaei et al.,^2^ and Ross et al.^3^ reported the first cases of the iatrogenic diversion of the IVC in low-lying ASDs but without associated anomalous IVC orifices or other congenital anomalies. Such cases would have been more frequent before the introduction of cardiopulmonary bypass because the patients used to be operated on via hypothermia and inflow occlusion. 

In the complete diversion of the IVC into the left atrium, the patient becomes cyanotic and hypoxic. In contrast, in partial diversion, factors such as the degree of the IVC orifice stenosis, displacement of the IVC into the left atrium, and the relief of pulmonary congestion may mask the acute sign and symptom of complete diversion.^4^ The surgeon repairing the ASD must always bear in mind the possibility that the inferior edge of the ASD is the valve of the IVC. This is precisely the point which we failed to take into account in the case of our patient. The error could have been avoided if the surgeon had taken the precaution of excising the IVC valve.^1^^, ^^2^ If the surgeon has any doubt about the anatomy, it is advisable to release the occlusion on the IVC for a moment to see where the blood enters the heart form the IVC.^3^ It then becomes possible to redirect the IVC orifice into the right atrium by suturing a pericardial patch onto the left atrium wall below the IVC orifice. When the anatomy is clearly understood, it is easy to make a new orifice for the IVC inside the right atrium by placing the pericardial patch below the IVC orifice by taking several bites in the lower part of the left atrial wall so as to form the medial half circle of the IVC orifice. From this point, the pericardial patch can be connected (by running suture) to the lateral and upper corners of the ASD.^5^ If the error is made by diverting the IVC into the left atrium, it is absolutely necessary to reoperate on the patient as soon as possible. During reoperation, the implanted pericardial patch must be replaced with a new one in the correct position so that the orifice of the IVC can be opened and redirected into the right atrium.^6^^-^^10^

Byork^1^ reported the death of a patient in whom the IVC was left draining into the left atrium for two days. Our patient survived the diversion of the IVC into the left atrium for 49 hours. Our patient had an ASD, where the IVC seemed to enter the left atrium and there was a large Eustachian valve, which was erroneously used to repair the defect. This resulted in the diversion of the IVC into the left atrium. To our knowledge, there are only a few similar cases of ASDs. Our thorough literature review yielded no cases of an early diagnosis of the diversion of the IVC accompanied by other congenital anomalies and such complications as severe hypoxia, perforated peptic ulcer, and hypovolemic cardiogenic shock. 

The mechanism of a perforated peptic ulcer is related to exposure to hypoxia, which is accompanied by increased leukocyte-endothelial cell adhesion. Adhesion occurs in microcirculation during the period of hypoxia and may persist. Systemic hypoxia may prime the microcirculation and increase the likelihood of gastrointestinal injury with other insults. The mechanisms by which acute hypoxia alters the interaction between the leukocyte and the endothelial cells remain to be determined. Often, no change in the total gastrointestinal blood flow is observed. However, because of the failure to augment the blood flow during hypoxemia, O_2_ delivery to the gastrointestinal tract can be decreased significantly. Despite this, the gastrointestinal O_2_ consumption is not compromised because tissue O_2_ extraction by the gastrointestinal tract rises significantly. Due to its low blood supply and high acid content, the duodenum is predisposed to the effect of hypoxia and perforation, as was the case in our patient. This is the first report of such a case in the existing medical literature.

## Conclusion

In our patient, our surgeon did not fully grasp the exact anatomy of this anomaly initially. Had the surgeon taken the precaution of following the direction of the IVC cannula tip into the left atrium, this complication would have been avoided. When in doubt about the anatomy, the surgeon should release the occlusion on the IVC for a moment to see where the blood enters the heart form the IVC and request intraoperative transesophageal echocardiography so as to be able to redirect the IVC orifice into the right atrium by suturing a pericardial patch onto the left atrium wall below the IVC orifice. Preoperative angiography is useful in detecting associated anomalies in cyanotic patients with the ASD. 

## Notes:

***This paper should be cited as: ***Sabzi F. Iatrogenic Diversion of Inferior Vena Cava into Left Atrium after Surgery for a Rare Combination of Congenital Heart Diseases. J Teh Univ Heart Ctr 2016;11(2):88-91.
